# Challenging Anticoagulation With Warfarin and Heparin Following Mechanical Mitral Valve Replacement in a Patient With Infective Endocarditis: A Case Report Highlighting Diagnostic Difficulties

**DOI:** 10.1002/ccr3.72695

**Published:** 2026-05-18

**Authors:** Musawer Khan, Ayesha Ahmad, Moin Khan, F. N. U. Zainullah, Nauman Khan, Fatima Nazir, Wakil Ahmed, Naqeeb Ullah, Muhammad Anees, Kamil Ahmad Kamil

**Affiliations:** ^1^ Combined Military Hospital Quetta Quetta Baluchistan Pakistan; ^2^ Khyber Medical University Peshawar Pakistan; ^3^ Quetta Institute of Medical Sciences Quetta Baluchistan Pakistan; ^4^ Rawalpindi Medical University Rawalpindi Pakistan; ^5^ Sandeman Provincial Hospital Quetta Pakistan; ^6^ Internal Medicine Department Mirwais Regional Hospital Kandahar Afghanistan

**Keywords:** anticoagulation failure, heparin resistance, infective endocarditis, mechanical mitral valve, pharmacogenetics, warfarin resistance

## Abstract

Warfarin remains the cornerstone of anticoagulation therapy following mechanical heart valve replacement. However, challenging anticoagulation responses, resistance to warfarin, and even more rarely to both warfarin and heparin pose significant clinical challenges and complicate management strategies. We describe a 44‐year‐old male with infective endocarditis who underwent mechanical mitral valve replacement. Post‐operatively, despite escalating warfarin doses up to 90 mg/day 15 mg/day, the patient's INR remained persistently subtherapeutic (maximum 1.67), his INR remained below therapeutic levels. A switch to low‐molecular‐weight heparin also failed to achieve adequate anticoagulation was initiated, but due to unavailability of anti‐Xa monitoring, its effectiveness could not be objectively assessed; clinically, there was evidence of ongoing thrombotic risk. Compliance, drug interactions, dietary influences, and malabsorption were considered and excluded. Pharmacogenetic testing and serum warfarin levels were unavailable locally. Definitive testing for pharmacogenetic mutations, serum warfarin levels, and antithrombin III activity was not available at our institution, representing key limitations. This case illustrates an extremely rare instance of dual anticoagulant resistance profound challenges in achieving therapeutic anticoagulation with two standard agents, highlighting the need for a structured diagnostic algorithm including compliance verification, pharmacokinetic and pharmacodynamic assessment, consideration of genetic testing (VKORC1, CYP2C9), and evaluation for antithrombin deficiency. Alternative vitamin K antagonists or direct oral anticoagulants may be considered in select refractory cases, although evidence for their use in mechanical valves is lacking. Alternative vitamin K antagonists (e.g., acenocoumarol) may be considered in refractory cases, but direct oral anticoagulants are strictly contraindicated in mechanical valves based on the RE‐ALIGN trial. Clinicians should maintain a high index of suspicion for challenging anticoagulant response resistance when INR remains subtherapeutic despite high doses and evaluate all possible etiologies systematically. This case expands current understanding of refractory anticoagulation following valve replacement and highlights the diagnostic limitations faced in resource‐constrained settings.

**Key Clinical Message**

Challenging anticoagulation responses to warfarin and heparin can occur after mechanical valve replacement. When advanced testing is unavailable, systematic exclusion of acquired causes (non‐adherence, drug interactions, diet) is essential. Multidisciplinary collaboration and individualized strategies are vital to prevent thromboembolic complications.

**Abbreviations**
aPTT
: Activated Partial Thromboplastin Time
AT
: Antithrombin
CECT
: Contrast‐Enhanced Computed Tomography
CYP2C9
: Cytochrome P450 2C9
CYP4F2
: Cytochrome P450 4F2
DOAC
: Direct Oral Anticoagulant
IE
: Infective Endocarditis
INR
: International Normalized Ratio
LMWH
: Low‐Molecular‐Weight Heparin
MR
: Mitral Regurgitation
NOAC
: Novel Oral Anticoagulant
PT
: Prothrombin Time
TEE
: Transesophageal Echocardiography
VKORC1
: Vitamin K Epoxide Reductase Complex Subunit 1

## 1 Introduction

Mechanical heart valve replacement is advised for various heart valve disorders in patients younger than 50 years old over bioprosthetic valves because of their durability. However, lifelong anticoagulation is necessary due to the elevated risk of thromboembolic disorders that can arise from the placement of this valve. Warfarin, an anticoagulant drug, is the primary choice to prevent these disorders, but its dosage needs to be altered based on the international normalized ratio (INR) due to the narrow therapeutic window and variability among patients [1].

In rare cases, either acquired or hereditary warfarin resistance has been noted, where the typical dosage fails to extend the prothrombin time (PT) or elevate the INR to the therapeutic range. The specified range to classify an individual resistant is over 105 mg per week (15 mg/day). Warfarin resistance is generally defined as the inability to achieve a therapeutic INR despite daily doses exceeding 15–20 mg [2]. A distinctive characteristic of this resistance is that only a small quantity of vitamin K is required to counteract the drug's effect [2].

To resolve the resistance, there are only two choices: either to raise the dosage of warfarin to attain the therapeutic range of PT and INR or use a different type of anticoagulant, such as subcutaneous heparins (unfractionated and low‐molecular‐weight heparins) [1, 2]. In this case report, an uncommon occurrence is addressed, wherein the patient exhibited resistance to a suboptimal response to both warfarin and heparin. When a patient requires more than 35,000 units of heparin within 24 h to sustain activated partial thromboplastin time (aPTT), they are deemed resistant. Heparin resistance is typically defined as the need for > 35,000 units of unfractionated heparin per day to achieve therapeutic aPTT, or failure to achieve adequate anti‐Xa levels with standard LMWH dosing [3]. Apparent heparin resistance is defined by an insufficient aPTT response, despite the preserved antithrombotic activity of heparin, as indicated by anti‐factor Xa levels. High levels of factor VIII are a common reason why people seem to be resistant to heparin. This occurs because they lower the in vitro aPTT without changing the in vivo antithrombotic effect of heparin [3].

## 2 Case Presentation

We report a case of a 44‐year‐old male, non‐smoker, permanent resident of Narowal, Punjab, Pakistan, with no comorbidities, admitted to the Hospital with chief complaints of fever and evaluation of anemia for the last 20 days. Fever was gradual in onset, high grade documented (102°F), intermittent, usually spiking at night, associated with rigors, chills, and productive cough, with no aggravating or relieving factors. There was no history of weight loss, loss of appetite, blood in urine, or joint and muscle aches. The patient had no recent travel history, trauma, or contact with sick individuals. Past medical, surgical, and family history was insignificant, and no drug intake or allergies were reported. A systematic review was unremarkable except for fever and cough.

On general physical examination, the patient was conscious, oriented, and mildly pale with pallor positive. His vitals were: heart rate = 80 bpm, blood pressure = 120/85 mmHg, temperature = 102°F, respiratory rate = 14/min, SpO2 = 96% on room air. Systemic examination was unremarkable.

## 3 Timeline of Clinical Events

**Table:** 

Date/Day	Event	Outcome
Day −20	Onset of fever and cough	Persistent fever
Admission	Baseline investigations and imaging	Splenomegaly, infarcts
Week 1	Blood culture positive for *A. baumannii*	Targeted antibiotics started
Week 2	Echocardiography confirmed severe MR	Surgery planned
July 2024	Mitral valve replacement	Successful surgery
Post‐op	Warfarin started and escalated	INR ≤ 1.67 despite 90 mg/day
Post‐op Week 2	Switched to LMWH (Clexane 120 mg daily)	No anticoagulant effect
Follow‐up	All causes explored (drug, diet, absorption)	Dual resistance confirmed

## 4 Diagnostic Assessment

### 4.1 Preoperative Evaluation

On admission, comprehensive baseline investigations were performed:

**Table:** 

Test	Result	Interpretation
Hemoglobin	9.3 g/dL	Anemia of chronic disease
White blood cells	13 × 10^9^/L	Leukocytosis
Platelets	280 × 10^9^/L	Normal
C‐reactive protein (CRP)	160.8 mg/L	Marked inflammation
Erythrocyte sedimentation rate (ESR)	88 mm/h	Elevated, consistent with infection
Albumin	27 g/L	Hypoalbuminemia (nutritional depletion/inflammation)
Prealbumin	12 mg/dL	Low, confirming nutritional deficiency
ALT	45 U/L	Mildly elevated
AST	38 U/L	Within normal limits
Total bilirubin	0.8 mg/dL	Normal
Alkaline phosphatase	92 U/L	Normal
PT	13.2 s	Normal
INR	1.1	Normal baseline
aPTT	32 s	Normal
Fibrinogen	645 mg/dL	Elevated (acute phase reactant)
Thyroid function (TSH)	2.1 μIU/mL	Normal
Free T4	1.2 ng/dL	Normal

Infectious work‐up, including dengue, malaria, Brucella, hepatitis B/C, and HIV, was negative. Blood cultures grew *Acinetobacter baumannii* sensitive to minocycline and colistin.

## 5 Imaging

Contrast‐enhanced computed tomography (CECT) of the abdomen showed hepatomegaly, splenomegaly with multiple hypodense splenic infarcts, and mild bilateral pleural effusions. Transthoracic echocardiography revealed a flail posterior mitral leaflet and severe mitral regurgitation. Transesophageal echocardiography confirmed posterior leaflet prolapse with ruptured chordae tendineae; no vegetations were visualized.

The diagnosis of infective endocarditis was established based on the modified Duke criteria (one major criterion: typical echocardiographic findings; three minor criteria: fever, predisposing heart condition, embolic phenomena).

## 6 Therapeutic Interventions

**Table:** 

Intervention	Details	Outcome
Empirical antibiotics	Ceftriaxone 2 g daily	No clinical improvement
Targeted antibiotics	Minocycline 100 mg BID + Colistin 9 MU loading then 4.5 MU BID	Infection resolved; repeat blood cultures negative at 2 weeks
Mitral valve replacement	Mechanical mitral valve replacement (St. Jude Medical 27 mm) performed on 27–6‐24	Successful surgery
Intraoperative details	Cardiopulmonary bypass time: 118 min; Aortic cross‐clamp time: 89 min; Initial ACT post‐heparin: 512 s (target achieved); Additional heparin bolus: 5000 units given; Transfusions: 2 units PRBCs intraop, 4 units FFP postop for coagulopathy	Uneventful bypass with adequate anticoagulation
Warfarin therapy	Initiated postoperative day 1; escalated progressively to 15 mg/day by week 3	INR remained ≤ 1.67 (maximum 1.67 at 15 mg/day)
LMWH therapy	Enoxaparin (Clexane) 1 mg/kg BID (120 mg/day) initiated when INR failed to respond	Anti‐Xa monitoring unavailable; clinically, splenic infarcts present preoperatively, no new thrombotic events documented
Compliance verification	All doses administered and documented by nursing staff during hospitalization	Non‐adherence excluded
Dietary assessment	Consultation with clinical nutrition; no excessive vitamin K intake identified	Dietary cause excluded
Drug interaction review	Complete medication review; no interacting drugs identified	Drug‐induced resistance excluded

**FIGURE 1:**
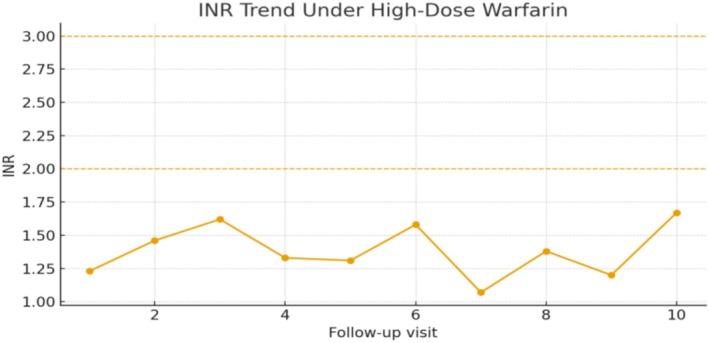
INR trend under high‐dose warfarin therapy after mechanical mitral valve replacement. Despite daily doses up to ~ ~90 mg ~ ~15 mg, INR values remained subtherapeutic and never reached the target range of 2.0–3.0; the maximum observed INR was 1.67.

Figure 1 demonstrates the persistent sub therapeutic INR despite escalating warfarin doses.

## 7 Investigations

**Table:** 

Test	Result	Interpretation
CBC	Hb 9.3 g/dL, WBC 13 × 10^9^/L	Anemia, leukocytosis
CRP	160.8 mg/L	Marked inflammation
ESR	88 mm/h	Marked inflammation
Albumin	27 g/L	Hypoalbuminemia
Fibrinogen	645 mg/dL	Elevated acute phase reactant
Blood cultures	*A. baumannii*	Infective endocarditis
CECT Abdomen	Splenic infarcts, hepatomegaly	Embolic complication
Transesophageal echo	Severe MR, flail leaflet	Surgical indication
Histopathology	Granulation tissue, no organisms	Confirmed healed IE
Antithrombin III activity	Not available	Limitation
Anti‐Xa assay	Not available	Limitation
Genetic testing (VKORC1, CYP2C9)	Not available	Limitation
Serum warfarin level	Not available	Limitation

## 8 Discussion

Warfarin remains the standard anticoagulant for oral use in adult patients after receiving prosthetic valve replacement; however, the roles of genetics, metabolism, and environment can tremendously impact the effectiveness of warfarin. Our patient showed persistent subtherapeutic INR on extremely high doses of warfarin (90 mg daily) and poor efficacy on therapeutic low molecular weight heparin (LMWH) at the same time. Dual resistance is an exceedingly rare occurrence and poses significant clinical dilemmas.

Warfarin remains the standard oral anticoagulant for patients with mechanical prosthetic valves. However, genetic, metabolic, and environmental factors can significantly impact its effectiveness. Our patient demonstrated a persistently subtherapeutic INR despite warfarin doses up to 15 mg/day, with a maximum INR of only 1.67. Concurrently, therapeutic‐dose LMWH was administered, but its effectiveness could not be objectively assessed due to the unavailability of anti‐Xa monitoring. This dual challenge in achieving anticoagulation is rare and poses significant clinical dilemmas.

Warfarin resistance is a rare phenomenon (< 0.1%), characterized by the requirement for warfarin to exceed 70 mg/week to maintain the INR within the clinically desired range [4]. Warfarin resistance may be acquired, often due to factors such as non‐adherence, drug interactions, excessive vitamin K intake, or malabsorption [5], or inherited, generally due to polymorphisms in the VKORC1, CYP2C9, and CYP4F2 genes, which affect metabolism or sensitivity to warfarin [6, 7]. In our patient, we systematically excluded acquired causes: adherence was ensured by directly observed therapy during hospitalization; a detailed dietary review revealed no excessive vitamin K consumption; and comprehensive medication reconciliation identified no interacting drugs. These exclusions point toward a possible inherited or intrinsic pharmacodynamics resistance, though genetic confirmation was unavailable.

Multiple reports exist of patients presenting with a similar resistance and similar challenges in those with prosthetic heart valves. In a case by Malik et al., a patient with a prosthetic mitral valve presented with a subtherapeutic level of subtherapeutic INR on warfarin doses up to 50 mg/day. The patient was noted to have mutations in both vitamin K epoxide reductase (VKORC1) and cytochrome P450 2C9 (CYP2C9), ultimately requiring reoperation to change from a mechanical to a bioprosthetic valve, thus eliminating the need for anticoagulation [8]. In a similar vein, Favaedi et al. reported a patient with mechanical prosthetic heart valves requiring warfarin doses of 100 mg/day without achieving target INR, yet no VKORC1 or CYP2C9 mutation was identified [9]. In a case from Ukraine, a patient with mechanical prosthetic heart valves had unexplained warfarin resistance, ultimately managed by switching to acenocoumarol [10]. These cases demonstrate both genetic and multifactorial characteristics of resistance mechanisms, but many of the contributing factors to these mechanisms remain poorly understood and elucidated. highlighting the heterogeneity of this condition.

## 9 Heparin Resistance

Interestingly, our case highlighted an inadequate anticoagulation response to LMWH. Our patient also demonstrated an apparent inadequate response to LMWH. Heparin resistance is classified as the failure to achieve a predictable anticoagulation effect, despite adequate dosing. Causes include antithrombin (AT) deficiency (congenital or acquired), increased heparin‐binding proteins, increased clearance, or systemic inflammation [11, 12]. Cardiac surgery and critical illness, both pertinent to our patient, are established risk factors for acquired heparin resistance [11]. The inflammatory state from infective endocarditis can further reduce heparin responsiveness due to elevation of acute‐phase proteins (e.g., factor VIII, fibrinogen) and consumption of natural anticoagulants [11, 12].

Importantly, in our patient, definitive confirmation of heparin resistance was not possible. Anti‐Xa monitoring—the gold standard for assessing LMWH effect—was unavailable. Additionally, antithrombin III levels could not be measured. The elevated fibrinogen (645 mg/dL) and inflammatory markers suggest that acute phase reactants may have contributed to an apparent heparin resistance, without true pharmacologic failure.

Similar instances of heparin resistance have been documented. A prospective study of patients undergoing cardiopulmonary bypass for infective endocarditis found that even patients with “stabilized” IE showed a significant reduction in heparin responsiveness, correlating with lower antithrombin‐III activity and higher fibrinogen levels [13].

### 9.1 Pathophysiological Considerations

An interesting pathophysiological duality was present in our patient. The significant hypoalbuminemia (27 g/L) would be expected to increase the free fraction of warfarin, potentially leading to an exaggerated INR response to standard doses. Paradoxically, we observed the opposite: a subtherapeutic INR despite high warfarin doses. Conversely, the acute inflammatory state would typically elevate factor VIII, potentially shortening the aPTT and creating the appearance of heparin resistance. This case underscores the complex and often unpredictable interplay between critical illness, inflammation, and anticoagulant drug response, where multiple opposing forces may be at play.

### 9.2 Therapeutic Implications

These findings exemplify the necessity of a structured framework when dealing with anticoagulant resistance failure. Discovering a warfarin‐resistant patient with an inadequate warfarin response leads to compliance evaluation, drug–drug interaction assessment, dietary vitamin K intake review, and malabsorption evaluation, along with pharmacogenetic testing if available [5, 6, 7]. Discovering a heparin‐resistant patient suspected of heparin failure would necessitate evaluation for antithrombin deficiency, measurement of AT activity, consideration of AT supplementation, or transitioning to an anticoagulant that does not require AT (direct thrombin or factor Xa inhibitors) [11, 12, 13].

For patients with mechanical valves, it is crucial to note that direct oral anticoagulants (DOACs) are strictly contraindicated. The RE‐ALIGN trial demonstrated increased rates of thromboembolic and bleeding events with dabigatran compared to warfarin in mechanical heart valve patients [14]. Owing to this, alternative management options exist but remain individualized. Alternative strategies that could be considered in similar resource‐limited settings include: (1) trial of a different vitamin K antagonist such as acenocoumarol or phenprocoumon, which have different metabolic pathways; (2) long‐term unfractionated heparin with antithrombin supplementation if deficiency is confirmed or suspected; (3) referral to a specialized pharmacogenomics or thrombosis center for advanced testing including warfarin genotyping and anti‐Xa monitoring; and (4) in extreme cases, multidisciplinary discussion regarding the risk–benefit ratio of maintaining a mechanical valve versus replacement with a bioprosthesis.

## 10 Strengths and Limitations

The value of this report is that it describes an extremely rare phenomenon: an unusual and challenging clinical scenario of combined resistance and suboptimal response to both warfarin and heparin, which provides a meaningful contribution to clinicians caring for anticoagulated patients with prosthetic heart valves. Placing our case in the context of prior documented cases, this adds to the limited but expanding cumulative evidence of anticoagulation resistance difficulties.

That said, our case was also limited. Several important limitations must be acknowledged:

- Anti‐Xa monitoring was unavailable, preventing definitive assessment of LMWH efficacy.
- Antithrombin III activity could not be measured, limiting evaluation for heparin resistance mechanism.
- Genetic testing for VKORC1, CYP2C9, and CYP4F2 polymorphisms was not performed, restricting identification of a specific warfarin resistance mechanism.
- Serum warfarin levels could not be obtained, which might have distinguished pharmacokinetic from pharmacodynamic failure.
- Factor VIII levels, which could have confirmed pseudo‐resistance to heparin, were not measured.

Genetic testing for VKORC1, CYP2C9, and CYP4F2 polymorphisms, along with antithrombin activity, was not performed, which restricts us from identifying the specific resistance mechanism. Also, with any single case report, the findings are not generalizable but certainly provide clinicians with a useful perspective as they encounter similar cases.

These limitations reflect the reality of clinical practice in resource‐constrained settings and highlight the need for increased access to specialized coagulation testing. Despite these limitations, the case provides valuable clinical insights for practitioners facing similar challenges.

## 11 Follow‐Up and Outcomes

Despite maximizing warfarin dosing and switching to LMWH, therapeutic anticoagulation was not achieved with warfarin. Compliance, diet, absorption, drug interactions, and laboratory error were systematically ruled out. As detailed above, acquired causes were systematically excluded. Pharmacogenetic testing and serum warfarin level measurement were not available locally. Multidisciplinary consultation recommended considering an alternative vitamin K antagonist (e.g., acenocoumarol) or referral to a specialized center for genetic testing and antithrombin activity assessment.

The INR trend shown in Figure 1 demonstrates the patient's persistent subtherapeutic anticoagulation despite high‐dose warfarin therapy. Over several months of follow‐up, INR values fluctuated between 1.07 and 1.62, never achieving the target therapeutic range of 2.0–3.0 recommended for mechanical mitral valve replacement. The maximum INR observed was 1.67, despite warfarin dosing as high as ~90 ~15 mg/day. This erratic and inadequate response, despite strict adherence and exclusion of drug–drug interactions, is indicative of true warfarin resistance. This response pattern suggests true pharmacodynamic resistance, though genetic confirmation was not possible.

## 12 Patient Perspective

The patient expressed frustration over repeated blood tests and lack of INR improvement despite taking high doses of warfarin. He appreciated the multidisciplinary approach and understood the need for specialized testing and individualized treatment.

## 13 Conclusion

We report a rare case of combined warfarin and heparin resistance challenging the case of subtherapeutic anticoagulation with both warfarin and LMWH following mitral valve replacement for infective endocarditis. This condition demands a structured diagnostic and therapeutic approach, including a thorough evaluation of compliance, absorption, genetic factors, and coagulation pathways. While definitive confirmation of true pharmacological resistance was limited by unavailable specialized assays, the case highlights important clinical lessons. Clinicians should consider individualized alternatives in refractory cases and collaborate with hematology and cardiology specialists for optimal management in resource‐limited settings where advanced testing is not accessible.

## Author Contributions

**Musawer Khan:** conceptualization, data curation, investigation, methodology, resources, supervision, writing – original draft, writing – review and editing. **Ayesha Ahmad:** conceptualization, methodology, writing – original draft. **Moin Khan:** resources, supervision, validation, writing – review and editing. **F. N. U. Zainullah:** methodology, writing – original draft. **Nauman Khan:** conceptualization, methodology, writing – original draft. **Fatima Nazir:** methodology, writing – original draft. **Wakil Ahmed:** writing – original draft. **Naqeeb Ullah:** resources, writing – original draft. **Muhammad Anees:** methodology, writing – original draft. **Kamil Ahmad Kamil:** conceptualization, methodology, writing – review and editing.

## Funding

The authors have nothing to report.

## Ethics Statement

All procedures performed during the study (case report) were in accordance with ethical standards of the faculty of Medicine, Institutional Ethical Review Board (IERB) CMH Quetta.

## Consent

Written Informed Consent was obtained from the participants for publication of this case.

## Conflicts of Interest

The authors declare no conflicts of interest.

## Data Availability

The data that support the findings of this case report are available from the corresponding author upon reasonable request.
